# The Impact of Curcumin on Immune Response: An Immunomodulatory Strategy to Treat Sepsis

**DOI:** 10.3390/ijms232314710

**Published:** 2022-11-25

**Authors:** Alessandro Allegra, Giuseppe Mirabile, Roberta Ettari, Giovanni Pioggia, Sebastiano Gangemi

**Affiliations:** 1 Division of Hematology, Department of Human Pathology in Adulthood and Childhood “Gaetano Barresi”, University of Messina, 98125 Messina, Italy; 2 Department of Chemical, Biological, Pharmaceutical and Environmental Chemistry, University of Messina, 98100 Messina, Italy; 3 Institute for Biomedical Research and Innovation (IRIB), National Research Council of Italy (CNR), 98164 Messina, Italy; 4 Allergy and Clinical Immunology Unit, Department of Clinical and Experimental Medicine, University of Messina, 98125 Messina, Italy

**Keywords:** curcumin, primary immunodeficiency, secondary immunodeficiency, sepsis, T cells, B cells, viral infections, bacterial infections, inflammatory immune response

## Abstract

Primary and secondary immunodeficiencies cause an alteration in the immune response which can increase the rate of infectious diseases and worsened prognoses. They can also alter the immune response, thus, making the infection even worse. Curcumin is the most biologically active component of the turmeric root and appears to be an antimicrobial agent. Curcumin cooperates with various cells such as macrophages, dendritic cells, B, T, and natural killer cells to modify the body’s defence capacity. Curcumin also inhibits inflammatory responses by suppressing different metabolic pathways, reduces the production of inflammatory cytokines, and increases the expression of anti-inflammatory cytokines. Curcumin may also affect oxidative stress and the non-coding genetic material. This review analyses the relationships between immunodeficiency and the onset of infectious diseases and discusses the effects of curcumin and its derivatives on the immune response. In addition, we analyse some of the preclinical and clinical studies that support its possible use in prophylaxis or in the treatment of infectious diseases. Lastly, we examine how nanotechnologies can enhance the clinical use of curcumin.

## 1. Introduction

### Immunodeficiencies and Infections

Sepsis is the penetration of microorganisms into the blood, with the stimulation of the host’s immune reaction against this invasion [1,2,3]. Various experiments have led to the definition of a scoring procedure for the precocious detection of organ failure as sepsis provokes a rapid stimulation of both pro- and anti-inflammatory responses which are capable of damaging different organs [4,5,6,7].

Although the commencement and progress of infection differs across patients, immunosuppression is reported in most infective subjects, and it seems to be notably related with prognosis [8]. The immune system is the greatest protection against pathogens, and with a correctly operative immune response, sepsis can be fully resolved [9]. It is therefore evident that a primary or secondary alteration in the immune response can lead to an increased incidence of infectious events and a worse prognosis.

The possibility of immune dysfunction should be considered in subjects with an augmented occurrence of infections, problematic healing, uncommon gravity, requirement of parenteral treatment, or unusual etiological agents. Moreover, there are different situations that disturb the immune response which may also indicate a causal alteration of the immune system. For instance, malnutrition alters the immune effectors, but it is also one of the characteristic symptoms of a subject affected by severe combined immunodeficiency [9].

The biological processes underlying an increased incidence of critical infections in apparently normal subjects is essentially undetermined, but epidemiological analyses indicate a possible relevant genetic influence [10]. It has been suggested that serious sepsis in children is more likely to be the sign of exceptional, single-gene alterations [11].

**Figure 1:**
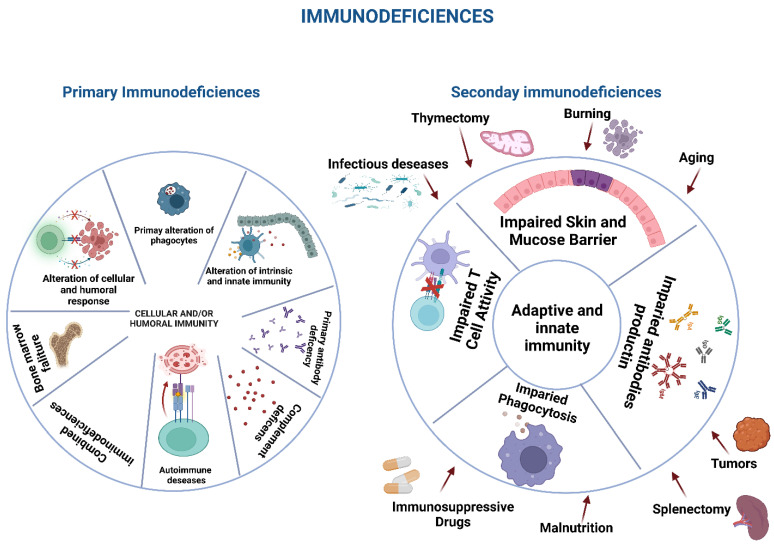
Different aspects of primary and secondary immunodeficiencies.

Different classifications have been employed in the last 50 years to define the different types of immunodeficiency, generally using the different forms of immune effector involved in the alteration. In any case, immunodeficiencies are distinguished as primary or secondary immunodeficiencies. Currently, the International Union of Immunological Societies recognizes over 400 primary immunodeficiencies (PID) classified into groups in relation to the type of cells involved and the underlying mechanism of immunological alteration [12,13,14,15,16,17] (Figure 1).

Secondary immunodeficiencies are transitory or permanent alterations of the immune response, provoked by elements that are not inherent to the same immune system. This class of agents comprises drugs, environmental factors, and a multiplicity of diverse situations. In any case, it is essential to evaluate the possibility of a secondary immunodeficiency as its incidence is more frequent than primary immunodeficiency [18].

Secondary immunodeficiencies are frequent due to taking medications used to treat diseases such as inflammatory diseases, autoimmune conditions, allergic pathologies, tumours, or graft-versus-host diseases. These medications can modify the cytokine production and cell function and include immunosuppressive small molecules such as steroids, cyclosporin, and methotrexate; protein kinase inhibitors and biological substances such as anti-CD20 antibodies; as well as old and new drugs such as bortezomib, cyclophosphamide, venetoclax, and idelalisib [19,20,21].

Major and massive trauma, severe scalding, and surgical interventions are also able to induce significant alterations in the immune response and can provoke severe sepsis, with a mortality rate of over 30 percent [22]. Lastly, surgical ablation of the thymus or the spleen have a profound effect on the immune response due to their fundamental action as lymphoid organs. Thymectomy in children causes T cell decrease, with more specific involvement of CD4 T cells than CD8 T cells [23]. There is a tendency for lymphocytes to preferentially mature towards effector T cells and a decreased variety of the T cell repertoire, indicating early ageing of the immune system [24], and an increased risk of sepsis or autoimmune diseases have been described after thymectomy [25].

However, it is now known that the same infection can determine an alteration in the immune response capable of leading to a worse evolution and prognosis of infection (Figure 1).

Sepsis generally evolves in two phases, although both stages can happen at the same time [26]. The early hyperinflammatory stage, also called the “cytokine storm”, is characterised by the overwhelming discharge of inflammatory substances by the innate immune system, which can damage cells and tissues [27]. Immediately after the onset of inflammation, the intensity of the immune response diminishes, provoking a hypoinflammatory condition. The immune effectors then show functional exhaustion and a reduction in lymphoid and myeloid cells, provoking a severe impairment of the immune system [28]. These changes render subjects susceptible to secondary infections, generally provoked by opportunistic hospital microorganisms such as viruses, fungi such as *Candida albicans* (8.5% of cases), or other pathogens such as *Acinetobacter baumannii* (22.2%), and *Pseudomonas aeruginosa* (10.3%). These secondary infections generally take hold 48 h after the primary infection, indicating that the immune unresponsiveness has reached its maximum peak. Other studies evaluating the occurrence of secondary infection on prognosis have demonstrated that opportunistic pathogens further increase in a later period (>15 days) of sepsis with respect to the initial period (<6 days) [29].

Various complex processes are implicated in the onset and progression of infection-correlated immune alteration, comprising metabolic changes, epigenetic influences, endotoxin tolerance, programmed cell death, and autophagy [30,31,32,33,34,35,36,37]. A typical case is the onset of endotoxin tolerance, expressed as a reduced delivery of inflammatory cytokines after a rechallenge of endotoxin or other factors [38]. Other studies have proved that epigenetic control is significantly implicated in the establishment of endotoxin tolerance as demonstrated by considerable changes in the transcription of genes coding deacetylase enzymes.

Sepsis is, thus, a situation where there is a pronounced stimulation of innate immunity along with the inhibition of the conventional T-cell-originated immune response and with an increase in regulatory T cells (Tregs). This, then, provokes the onset of a sepsis-correlated immunosuppression [39,40,41] and leads to a disturbance of the immune response and possible subsequent organ damage [42,43].

Lastly, an important aspect that clarifies some of the characteristics of the genesis of immune system modifications in subjects affected by sepsis concerns the role of inflammasomes in the sepsis-correlated inflammatory response. Inflammasomes are a caspase-stimulating complex involving essentially caspase-1, caspase-5, Pycard/Asc, and NALP1 [44]. Inflammasomes seem to play a key role in regulating the immune response against microorganisms. For instance, inflammasome-defective animals harbour an abnormal microbial population which can be passed on to normal mice [45].

In contrast, the stimulation of inflammasomes induces inflammatory cell death through various procedures comprising necroptosis and pyroptosis, which can trigger an inflammatory immune response [46,47,48,49,50,51].

## 2. Curcumin and Infections

Sepsis is still a key cause of illness and death, thus highlighting the need for continued research to find new strategies and treatments [52]. Interventions regarding the primary or secondary alterations of the immune system that determine the onset, course, and prognosis of infection are fundamental for patients with sepsis. An enormous number of plants have been reported to be effective in vitro against a multiplicity of infections and bacteria [53].

The aim of our review was to evaluate the possible use of turmeric and its derivatives in the treatment of infections. Particular attention was paid to its ability to significantly alter the immune response and trigger increased resistance to infections.

Turmeric (*Curcuma longa* L.) is a perennial and rhizomatous herb belonging to the Zingiberaceae group and is an important medicinal plant [54]. Curcumin (Curcumin (1,7-bis(4-hydroxy-3-methoxyphenyl)-1,6-heptadiene-3,5-dione)) is a polyphenolic substance with hydrophobic features which is one of the most effective components in turmeric extract [55]. Curcumin can operate in several different signalling pathways, and, thus, dietary turmeric or curcumin demonstrate gastro-protective, antioxidant, anti-inflammatory, antitumour, and immune-modulatory actions [56,57]. Curcumin has also shown significant antibacterial and antiviral effects [58,59,60,61], and its tolerability and non-toxicity even at high dosages have been well demonstrated by clinical trials [62,63].

### 2.1. Curcumin and Its Effects on the Immune System

The immune controlling system is based on regulatory cells, such as regulatory B cells (B regs) and regulatory T cells (Tregs), and on a series of humoral regulators with an inhibiting or stimulating character, such as IL-10 or transforming growth factor (TGF)-β [64,65]. Curcumin cooperates with various cells that modulate the immune response, such as macrophages, dendritic cells, and B and T cells, as with interleukins and gene transcription elements [66,67].

The immunomodulatory action of curcumin occurs both through Treg inhibition, and stimulation of the effector T cells. One study showed that curcumin use on myeloid-derived suppressor cells [68] led to an increase in CD8 + T cells and a decrease in Treg cells, thereby boosting the immune response [69]. The effect of curcumin in stimulating immunity has also been demonstrated in numerous in vivo clinical trials, as curcumin administration increased Th1 cells by stimulating the Treg cell switch to Th1 cells and significantly decreased Treg cells via Foxp3 inhibition and the increased production of IFN-γ [70,71].

Curcumin administration to patients led to a decrease in inflammation and allergies and enhanced the innate immunity against tumour cells, cardiovascular diseases, and pathogens. Curcumin operates via the regulation of the gene expression of inflammatory cytokines and, thus, can decrease the intracellular activity of MAPKs, NF-κB, JAKs/STATs, and the Notch-1 pathway [72,73].

The action of curcumin on the immune system may also be effective in moderating age-related immunological changes. Although elderly subjects are not immunodefective, they do not have an optimal response to immunization, and consequently, strategies are needed to render them immunologically receptive. Programmed cell death 1 (PD-1), an inhibitory receptor, has a key effect in the control of autoimmune diseases, tumours, and infective diseases. Its presence in T cells suggests their functional exhaustion. One study demonstrated that curcumin decreased the rate of PD1 + cytotoxic T cells in elderly animals. Curcumin could thus be employed to enhance the immunological framework of elderly subjects, perhaps preserving them from infectious events [74].

Curcumin appears to influence other cell signalling molecules, such as apoptotic proteins, IKKβ, endothelin-1, C reactive protein, prostaglandin E2, GST, VCAM1, phosphorylase kinase, and HO1, many of which are able to modify the function of the immune system [75,76,77,78,79,80].

The above results were confirmed with a group of neoplastic patients particularly affected by immunological alterations. As in immunosuppressive cancers, curcumin reduced the programmed cell death of T cells, augmented the population of memory cells, and inactivated the effects of Treg cells, thus efficaciously overturning the influence of immunosuppressive cancer [81]. For instance, a new nanocurcumin increased the presence of co-stimulatory molecule CD86 on the membrane of dendritic cells and reduced the concentration of inflammatory substances produced by effector T cells [82]. One in vitro experiment also showed that small dosages of curcumin increased the population of CD8 + T cells and improved their IFN-γ generation [83].

Curcumin is also able to intervene in natural killer (NK) cells. One study evidenced that curcumin increased CD16 + and CD56dim in the membrane of NK-92 cells [84]. The action of curcumin in stimulating the cytotoxic ability of NK cells has also been correlated with the stimulation of STAT4 and STAT5 pathways in NK cells and the inhibition of pERK and PI3K generation in curcumin-treated breast cancer cells [84].

The effect of curcumin on NK cells was confirmed in a study of pancreatic tumours. In the experiment, curcuminoids improved the capability of NK cells to produce IFN-γ and stimulated the antitumour effect of NK cells against pancreatic cancer cell lines [85]. In addition, curcumin, to some extent, halted the tumour-exosome-mediated block of NK cell stimulation, which is due to the alteration of the ubiquitin–proteasome system. In fact, the treatment of animal breast tumour cells with curcumin led to an increase in ubiquitinated exosomal proteins with respect to untreated cells. Exosomes extracted from cancer cells treated with curcumin also showed less inhibition of IL-2-caused NK cell stimulation. These results confirm the importance of the effects of curcumin on NK cells [86].

### 2.2. Other Immune-Mediated Effects of Curcumin on Infections

Several other new mechanisms have been identified to explain curcumin’s effects on infections. Lipopolysaccharide (LPS), the main structural component of the Gram-negative bacteria membrane, is recognized as a central mediator of infection, as a disproportionate LPS can provoke septic shock [87]. LPS joins TLR4 and sends signals via transcription elements to generate an inflammatory condition. This phenomenon is due to the generation of specific cytokines, such as IL-6, TNF-α, and IL-1β, which attract innate and adaptive elements of inflammation to infection sites [88].

It has also been demonstrated in animal experimental models that curcumin can protect against sepsis-caused muscle proteolysis and acute lung damage [89,90,91,92]. The principal effect is probably an intense inhibitory action on inflammatory elements such as activator protein-1, cyclooxygenase, and inducible nitric oxide synthase [93].

Interestingly, the immunomodulated efficacy of curcumin can be enhanced through its administration. A transgenic animal experimental model was employed to control IL-1β generation in LPS-caused sepsis in order to evaluate the defence action of curcumin-loaded solid lipid nanoparticles (SLNs). Curcumin-SLNs were administered intraperitoneally before the intraperitoneal release of LPS. Curcumin-SLNs can decrease concentrations of IL-1β expression particularly three hours after LPS administration. Curcumin-SLNs also significantly reduce the expression of IL-6, TNF-α, and IL-1β, and increase the expression of the anti-inflammatory cytokine IL-10. A clear reduction in the sepsis-caused injury to organs such as heart, kidney, and liver was reported after curcumin-SLNs administration. The findings also demonstrated that curcumin-SLNs reduced the expression of TLR4, TLR2, and TNF-α in lymph nodes [94].

A different mechanism explaining the effects of curcumin on the immune system may be the action on the redox system. In fact, curcumin reduces oxidative-stress-correlated inflammation through phosphatidylinositol 3-kinase (PI3K)/AKT- and NF-κB-correlated pathways, thus reducing LPS-provoked sepsis and liver malfunction [95,96,97].

Another in vivo study, also performed in an animal experimental model, evaluated the consequences of dietary curcumin nanoparticle (C-NP) administration on the antioxidant condition and humoral immunity of Nile tilapia (Oreochromis niloticus) [98]. The fish were given food supplemented with C-NPs for 60 days. The number of red blood cells, leukocytes, haemoglobin, and haematocrit levels were significantly greater in treated fish than in the control group. Similarly, antioxidant components such as catalase, glutathione peroxidase, malondialdehyde, and superoxide dismutase, and humoral immunity evaluated as lysozyme production and total immunoglobulins were significantly greater in C-NPs-fed fish. This suggests that the administration of 45–55 mg/kg of C-NP can be used to enhance antioxidant factors and the immune response [98].

Lastly, curcumin also acts on non-coding genetic material. MicroRNA (miRNA) is a class of short single-stranded non-coding RNAs consisting of 18–22 nucleotides. This genetic material does not have the capacity of protein coding; however, it regulates gene expression by inhibiting the transcription of its target mRNA [99].

Several studies have confirmed that miRNAs have an essential effect in the onset and progress of infections. For instance, miRNA-133a exacerbates the inflammatory condition induced by sepsis through affecting sirtuin-1 [100]. On the other hand, augmented miRNA-223 expression increased the M2 macrophage population and reduced LPS-caused sepsis through alterations in glycolysis [101].

**Figure 2:**
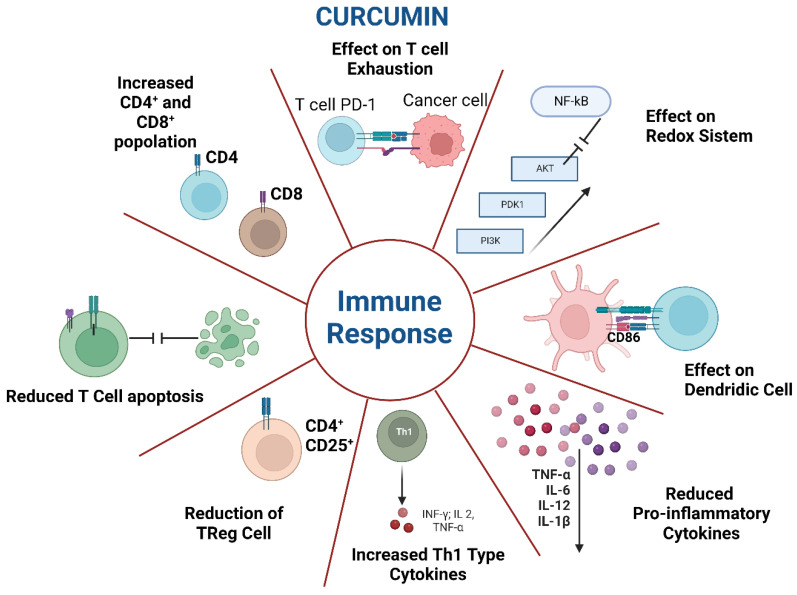
Effects of curcumin on immunological effectors.

Curcumin might function by regulating miRNA expression in the inflammatory response [102]. One study investigated the effects of curcumin on the immune response of infected animals through the increase in the miRNA-183-5p- and Cathepsin B (CTSB)-mediated phosphatidylinositol 3-kinase (PI3K)/AKT pathway [103]. Curcumin was administered for forced feeding, and i.v. administration of plasmid vectors of interference with miRNA-183-5p or CTSB was carried out. To induce sepsis, intraperitoneal administration of LPS was performed. Curcumin administration reduced tissue injury, decreased the number of inflammatory elements, and reduced the frequency of CD39 + Tregs in the venous blood of sepsis animals. Curcumin accelerated miRNA-183-5p, which negatively regulated CTSB and the curcumin-mediated PI3K/AKT pathway through the miRNA-183-5p/CTSB axis in order to limit inflammation and increase its immune activity [103] (Figure 2).

## 3. Curcumin and Virus Infections

This section provides a few examples of the results obtained in in vitro and in vivo experiments. Our aim is to demonstrate the anti-infective effects of curcumin against viral and bacterial infections via immunological and non-immunological activities. This section does not claim to be exhaustive but highlights the possible effects of curcumin in the most frequent or important viral or bacterial infections.

### 3.1. Curcumin and Human Immunodeficiency Virus

The acquired immunodeficiency syndrome (AIDS) is provoked by the human immunodeficiency virus (HIV), which inhibits the immune system. Since the 1980s, 75 million people have contracted this infection, with 32 million deaths. It remains one of the most frequent causes of death in Africa, where about 4% of the inhabitants are infected [104].

There are two different forms of HIV: HIV-1, which is responsible for the diffusion of the epidemic, and HIV-2 which is essentially confined to Africa [105,106]. The damaging action of this retrovirus is due to its ability to impact CD4 T cells, which stimulate the adaptive immune response [107], as different mechanisms of the virus can kill this type of cell.

The current therapy for HIV infection is based on anti-retroviral treatment. However, an old study evaluated the efficacy of curcumin in AIDS subjects but found no significant effects on viral growth. CD4 cells showed a small increase in patients with a high-dosage of curcumin with respect to the significant reduction registered in the low-dose group. In any case, curcumin was shown to enhance the well-being of most participants [108].

Curcumin likely has anti-HIV action by operating as an inhibitor of gp120 binding, and of protease, integrase, and topoisomerase II functions [109,110]. Curcumin loaded with apotransferrin capsulated in NPs connect to transferring receptors, provoking cell absorption and T cell toxicity, finally blocking HIV proliferation, and repressing the production of topoisomerase II [111].

Other studies have investigated other mechanisms of the action of curcumin. Enzyme HIV-1 integrase integrates the HIV virus DNA to further reproduce. Specific software has been used to run docking experiments, providing information on curcumin interactions and showing that it joins with the HIV integrase, thus blocking the proliferation. Curcumin also combines with the functional site that links the catalytic residues adjacent Asp and Asp and near to Mg2 + ion causing in integrase inhibitory effect against HIV [111]. A study evaluating two curcumin analogues, dicaffeoylmethane and rosmarinic acid, showed that both substances blocked the integrase activity [112,113].

Curcumin presents other mechanisms of action against HIV. HIV-1 gene expression is determined by Tat and Rev proteins, which stimulate the transcription and facilitate the transfer of mRNA which code viral proteins [114]. Curcumin blocks Tat protein, decreasing HIV proliferation. One study showed that curcumin (10–100 nM) blocked Tat stimulation of HIV-1-long terminal repeats (LTR), 80% in HeLa cells. It might thus work as an important substance in the combined treatment of HIV [115].

Curcumin is also a specific inhibitor of in vitro and in vivo p300/CREB-binding protein (CBP) histone acetyltransferase (HAT) activity, and curcumin also blocks the p300-mediated acetylation of p53 in vivo. It specifically represses the p300/CBP histone acetyltransferases activity-dependent transcriptional activation from chromatin. Curcumin also blocks the acetylation of HIV-Tat protein in vitro by p300 as well as the growth of the HIV, as demonstrated by the decrease in syncytia generation after curcumin administration in SupT1 cells [116].

As reported above, curcumin might operate via an effect on oxidative stress. Curcumin and 15 structural analogues with different operative groups combined with their aromatic rings were evaluated in terms of their antioxidant effects in vitro. The findings suggested that both curcumin and its analogues were powerful antioxidants, capable of removing free radicals. Compound 3e presented the greatest antiradical ability, while 3d and curcumin showed a higher antioxidant effect. Compounds such as 2d and 3d and curcumin have been shown to manage and reduce oxidative stress. Interestingly, compound 2e also showed the highest in vitro HIV-1 protease repressing effects [117].

An ongoing clinical trial is currently evaluating the effect of curcumin supplementation in patients with HIV/AIDS (NCT03141918: Supplementation of Bioactive Compounds on the Energy Metabolism of People Living With HIV/AIDS) [118].

### 3.2. Curcumin and SARS-CoV 2 Infection

COVID-19 is an infection provoked by RNA betacoronavirus which is related to SARS-CoV [119,120]. COVID-19 produces intense changes in the effector cell responsible for the immune response [121,122,123].

Curcumin produces its immune-enhancing action via free radical deactivation and through an improvement in the antioxidant system [124]. Curcumin regulates the inflammatory response to infection via the neutralisation of inflammatory transcription elements, such as the nuclear factor kappa B, signal transducer and activator of transcription 3, and induces the decreased expression of inflammatory cytokines [125,126]. In addition, for the duration of SARS-CoV-2, curcumin blocks the angiotensin-converting enzyme 2 production, which is necessary for virus entry. Curcumin thus stimulates anticoagulation and fibrinolysis and prevents critical COVID-19 [127,128,129,130].

The action of curcumin on the immune system may also be useful for effective immunisation against COVID-19. After vaccine administration, the antigen present stimulates B cells to produce IgM antibodies, the first antibody to be produced, and IgG, a more efficient neutralizing antibody [131]. The quantity of B cells generating antibodies may be T cell-dependent or -independent [132,133]. The action of curcumin in enhancing antibodies generated after COVID-19 vaccination has been studied [134]. A group of patients received a curcumin supplement after the first administration of the vaccine for a period of four weeks after the second vaccination. The number of antibodies against SARS-CoV-2 was evaluated four weeks after the second vaccination. The number of antibodies generated in the patients receiving the curcumin supplement was significantly higher compared to the control group. None of the variables examined (sex, age, and comorbidities) were shown to influence the generation of antibodies within the two groups [134]. The potential of curcumin was thus demonstrated for treatment during the period of vaccination.

Finally, a clinical trial has verified the efficacy of curcumin for precontact prophylaxis of COVID-19. This ability may be due to antiviral effects, such as an effect on viral membrane proteins, an alteration of viral envelope, inhibition of viral protease, and induction of antiviral response by improving immune response. Curcumin is also able to prevent severe pneumonia, probably by acting on the IL-6 trans signal, HMGB1 system, and NF-kB, and is well tolerated in infected patients [135]. In a systematic review performed on six randomized trials on 558 subjects, curcumin as an adjunct treatment in COVID-19 infected subjects improved clinical prognosis and decreased hyperinflammation [136].

### 3.3. Curcumin and Enterovirus

Enterovirus 71 (EV71) is a single-stranded RNA virus which is part of the genus Enterovirus, family Picornaviridae [137]. Although EV71 is usually asymptomatic or causes only mild symptoms, such as sore throat or fever [138], it may also cause encephalitis and pulmonary oedema. EV71 epidemics are a serious problem particularly in the Asia-Pacific region [139,140]. Furthermore, as there is no specific antiEV71 treatment, therapy is only supportive [141].

One study reported that curcumin displayed powerful antiviral action against EV71. Employing Vero cells infected with EV71, a supplementation with curcumin considerably reduced the production of viral RNA, the synthesis of viral protein, and the viral proliferation. In line with the previous reports for other viruses, curcumin decreased the generation of ROS caused by viral infection. However, the antioxidant ability of curcumin may not actually have participated in its antiviral effect, as N-acetyl-L-cysteine, a powerful antioxidant, failed to reduce viral growth. In any case, curcumin did decrease the activity of proteasomes, which was augmented by infection and increased the number of short-lived proteins, such as p53 and p21. The same study evaluated other possible antiviral effects of curcumin by evaluating the production of GBF1 and PI4KB, which are essential for the generation of the viral replication complex. The authors demonstrated that curcumin decreased the production of both proteins and reported that curcumin had an effect on programmed cell death at the initial phase of viral infection [142].

### 3.4. Effect of Curcumin on Other Viral Infections

As for the effects of curcumin on Hepatitis B infection, an experimentation positioned curcumin in lipid vesicles and dispensed them to transgenic mice with hepatitis B virus-related hepatocellular carcinoma [143]. This specific nutritional treatment decreased the size of tumours. Moreover, curcumin inhibited mTOR gene expression and was suggested as chemopreventive in subjects with chronic HBV [143].

Furthermore, curcumin reduces hepatitis C virus (HCV) gene expression through inhibition of the Akt-SREBP-1 stimulation. Importantly, curcumin has an immune-mediated effect on HCV, as the combined administration of curcumin and IFN alpha seems to produce a strong inhibitory action on HCV proliferation. Curcumin appears to reduce HCV proliferation in vitro and might, theoretically, be effective as a new anti-HCV agent [144]. In addition, curcumin can decrease the inflammation caused by the virus, regulating the production of inflammatory cytokines such as IL-4, 6, 8, and TNF-α and increasing the production of anti-inflammatory substances such as soluble intercellular adhesion molecule-1 and IL-10 [145].

Curcumin pretreatment of the virus decreased viral infectivity without lysing the virus. Experiments on membrane fluidity showed that curcumin changed the fluidity of the HCV envelope, impairing viral binding and fusion. Additionally, it has been discovered that curcumin prevents cell-to-cell transmission and works well when combined with other antiviral medications [146].

Finally, some studies were conducted to evaluate the possibility that the administration of curcumin can modify the pharmacokinetics and efficacy of some antivirals. A new oral medication called daclatasvir is being developed to treat persistent Hepatitis C Virus infections. This is a substrate for the primary pharmacokinetic interaction that involves CYP3A4 and P-glycoprotein. Wistar rats were used in pharmacokinetic studies of daclatasvir after oral dosing in the presence or absence of curcumin. According to those studies, curcumin pretreatment for seven days at a high dose level resulted in a much lower plasma level of daclatasvir than did concurrent single dose administration. It can be inferred that dose adjustment is unlikely to be necessary for intermittent use of curcumin at a low dose, but caution should be taken for chronic and concurrent use of curcumin at a high dose [147].

In an experimental animal model, curcumin was effective against herpes simplex 2 as well as papilloma (HPV) and Epstein Barr viruses in vitro [148]. It appears to inhibit transcription activation by the protein AP-1, causing a reduction in human T cell leukaemia virus (HTLV-1) and HPV-caused cellular transformation [149]. An in vitro effect against Friends leukaemia [150] and Newcastle and poliomyelitis viruses [151] has also been described.

Another study investigated curcumin’s efficacy against human adenovirus (HAdV). In infected A549 lung adenocarcinoma cells, curcumin induced a dosage-dependent reduction in production of the viral early 1A proteins, which are essential to complete the replicative cycle of the virus [64,65], suggesting that curcumin acts against different forms of HAdV. Curcumin administration also reduced the number of copies of the HAdV-5 viral genome and shortened the recovery period from the virus [152]. Nevertheless, the most efficient levels of curcumin were only a little smaller than the CC50 of curcumin, suggesting that curcumin has only a very limited curative window against HAdV [153] (Table 1).

## 4. Curcumin and Bacterial Infection

Besides its antiviral action, curcumin also intervenes in bacterial infections. Recurrent respiratory tract infections (RRTIs) are frequent infections in children and the anti-inflammatory substances and antibiotics employed to treat these infections frequently have severe side effects and participate in the onset of drug resistance. A report evaluated the immunologic effects of an oral administration of lactoferrin and curcumin (LC) in children with RRTIs [154]. Dispensation of LC was correlated with a significant and positive immune modulation and a decrease in RRTI occurrence in the children treated [155].

The effects of curcumin were also evaluated in patients with chronic bacterial prostatitis (CBP). CBP is an enduring infection of the prostate gland provoked by both Gram-negative and Gram-positive bacteria. Current therapies for CBP use antibiotics that pass through the prostate and destroy the pathogens. Nevertheless, the limited ability to penetrate the prostate tissue, the onset of drug resistance, and the collateral effects due to the use of antibiotic therapy mean that alternatives are needed.

A long-term follow-up analysis assessed the effectiveness of combined administration of Serenoarepens plus Urticadioica and curcumin plus quercetin in enhancing the efficiency of prulifloxacin in subjects with CBP [156]. A group of CBP patients were treated with prulifloxacin daily for 14 days, while a second group of patients were treated with a combined treatment of Serenoarepens plus Urticadioica and curcumin plus quercetin and antibiotics. One month after therapy, 89.6% of the subjects in the first group registered no clinical signs correlated with CBP, while only 27% of the subjects treated with prulifloxacin alone were free of symptoms. Furthermore, six months after therapy, no subjects in the first group had a relapse of infection, while some subjects in the second group did. Although curcumin seemed to increase the effectiveness of antibiotics in subjects with CBP [156], the absolute effect of curcumin in reducing CBP clinical signs was not determined.

## 5. Conclusions

Although curcumin certainly acts against infectious diseases by operating with both immunological and extra-immunological mechanisms, several obstacles prevent it from having wide clinical use. As with other substances of natural origin, the main difficulty is the drugability of its metabolites. Other issues are the authentication of plant substances and difficulties in the extraction and isolation procedures [157,158].

Despite all the advantages of curcumin, there are several drawbacks to its clinical employment such as chemical instability, bad water solubility, fast elimination, and limited assimilation [159].

Fortunately, new drug transport systems and the use of nanoparticles should make its therapeutic use possible. In fact, compared to conventional curcumin, curcumin linked with nanoparticles has a better dispersion in water and improved assimilation [160]. Moreover, the nanosized substances rest in the blood longer than traditional curcumin and improve its bioavailability [161].

Numerous novel transport and release methods have been explored in an effort to improve the anti-infectious efficacy of curcumin. For instance, a muco-inhalable delivery system (MIDS) loaded with silymarin can be used to overcome COVID-19 infection [162].

Moreover, according to a study, highly targeted and effective drug accumulation in the brain is made possible by nanomedicines created by functionalizing RVG, a neurotropic polypeptide produced from the rabies virus, and loading reduction-sensitive nanomicelles (polymer and doxorubicin) [163]. Curiously, curcumin inhibits the main efflux proteins in doxorubicin-resistant glioma cells despite acting as the hydrophobic core of the polymer. The RVG-modified micelles exhibit improved cell entrance and anticancer activity, according to studies on doxorubicin-resistant rat glioma cells. Research conducted in mice in vivo further demonstrated that RVG-modified nanomicelles greatly increased brain accumulation and tumour inhibition rate, resulting in a higher survival rate with less systemic damage. After the RVG-modified nanomicelles treatment, excellent suppression of recurrence and lung metastatic nodules was also identified.

A different technique involves the use of proniosomes which are the provesicular form of niosomes and can be made as either a liquid crystalline gel or a free-flowing powder by coating a hydrophilic carrier with a non-ionic surfactant. Using Design-Expert software, curcumin-loaded proniosomes were created using the slurry approach to show how different independent variables affect entrapment effectiveness and the percentage of medication released after 12 h (Q12h). After reconstitution, the improved formula (F5) showed good flowability and took the form of spherical nanovesicles [164]. Compared to the equivalent niosomes, F5 showed greater stability and a considerable increase in Q12h. F5 had much stronger antiviral efficacy and safety than curcumin. Acyclovir’s effectiveness dose was lowered by F5 while maintaining its safety, which resulted in a 100% decrease in viral plaques.

An interesting aspect is the possibility of using viral derivatives for the delivery of drugs. Virosomes are reconstituted viral envelopes consisting of viral glycoproteins and membrane lipids resembling the original virus but lacking any genetic material, making their internal compartment empty. The efficient drug delivery via virosomes can be ascribed to their nature of mimicking the natural way of infection by any virus, enabling them to specifically bind with target cell surface receptors for their entry inside the cell. Influenza virosomes were prepared by solubilisation of the viral membrane with 1,2-distearoyl-sn-glycerol-3-phosphocholine. During membrane reconstitution, the hydrophilic nanocurcumin was added to the solvent system, followed by overnight dialysis to obtain nanocurcumin (NC)-virosomes. The same was characterised using a transmission electron microscope and scanning electron microscope; a 3-(4,5-dimethylthiazol-2-yl)-2,5-diphenyltetrazolium bromide (MTT) assay was used to evaluate its in vitro cytotoxicity using MDA-MB231 and mesenchymal stem cells [165]. The results showed NC-virosomes have spherical morphology with size ranging between 60 and 90 nm. It showed an 82.6% drug encapsulation efficiency. The viability of MDA-MB231 cells was significantly inhibited by NC-virosome in a concentration-dependent manner at a specific time. The IC50 for nanocurcumin and NC-virosome was 79.49 and 54.23 µg/mL, respectively. The site-specific drug-targeting, high efficacy, and non-toxicity of NC-virosomes proves their future potential as drug delivery vehicles.

Similarly, an oil in water nanoemulsion coated with gH625—a membranotropic peptide derived from the glycoprotein H of Herpes simplex virus 1—was the basis for the nanocarrier that Fotticchia et al. proposed. Curcumin molecules are certainly directly incorporated into the cytosol rather than lysosomes when placed into nanocapsules. Finally, by examining the anti-inflammatory capabilities of the suggested nanocarrier, the authors demonstrated encouraging preliminary in vivo results [166].

New nanotechnologies can also be exploited. In fact, although in several tests curcumin has been combined with metal nanoparticles, particularly with gold particles (Au NPs) due to their modest toxicity, gold quantum clusters (Au QCs) composed of fewer atoms seem to have a better effect due to their specific physical characteristics. For example, Chen et al. generated lysozyme-protected Au QCs which displayed a greater antimicrobial effect than traditional gold nanoparticles [167].

Note that although nanocurcumin has a beneficial effect in the treatment of infective diseases such as HIV, the correct dose is still to be ascertained, and clinical trials should verify the results of numerous preclinical studies.

Furthermore, some investigators have described undesirable side effects correlated with this substance. Lao et al. performed a dose-escalation analysis to establish the maximum tolerable dosage and safety of a single oral administration of curcumin in healthy patients [168]. Only a few patients were subjected to insignificant toxicity that did not seem to be dose-correlated. The more frequent symptoms were rashes, diarrhoea, yellow stools, and headaches. In another study, curcumin administered for four months at dosages oscillating between 0.45 and 3.6 g/day caused nausea and diarrhoea and increased the serum lactate dehydrogenase and alkaline phosphatase levels [155].

In the future, other aspects of the correlations between curcumin and infections will need to be explored. Curcumin could also find use in the prevention of the toxic effects of antibiotics [169].

However, the analysis of the immunological effects exerted by curcumin remains the cornerstone of its use in infectious diseases. The study of its action on unconventional immune accessory cells [170] will hopefully lead to new inputs for the use of curcumin in patients with sepsis and disorders of the immunological effectors.

## Figures and Tables

**Table 1:** Modification of the immune response induced by curcumin towards viral infections.

Virus	Effect	Type of Study	Ref.
HIV	Increase in CD4 + cells. Greater perception of well-being.	In vivo	[108]
	Effects on gp120 binding, integrase, topoisomerase II. Reduced HIV proliferation.	In vitro	[109,110,111,112,113]
	Effect on Tat and Rev proteins. Reduced HIV proliferation.	In vitro	[114,115]
	Effect on p300CREB-CBP histone acetyltransferases.	In vivo and in vitro	[116]
	Effect on oxidative stress and HIV-1 proteases.	In vitro	[117]
SARS-COV 2	Free radical deactivation.	In vitro	[124]
	Effect on inflammatory transcription elements, reduced expression of proinflammatory cytokines.	In vitro	[125,126]
	Enhanced antibodies production after vaccination.	In vivo	[134]
	Effect on IL-6 trans signal, NF-kB. Prevention of severe pneumonia.	In vivo	[135]
Enterovirus	Reduction of proteasome activity. Effect on p53 and p21 protein.	In vitro	[144]
HCV	Reduction of inflammatory cytokines (IL-4, IL-6, IL-8, TNF. Increased expression of IL-10 and soluble intercellular adhesion molecule 1.	In vitro	[145]

## Data Availability

Not applicable.
